# Editorial: Training optimization through the recording of neuromuscular variables

**DOI:** 10.3389/fspor.2024.1479611

**Published:** 2024-08-28

**Authors:** Danica Janicijevic, Ivan Jukic, David Ulloa-Diaz, Amador García-Ramos

**Affiliations:** ^1^ Faculty of Sports Science, Ningbo University, Ningbo, China; ^2^ Department of Radiology, Ningbo No. 2 Hospital, Ningbo, China; ^3^ Department of Sports Sciences and Physical Conditioning, Faculty of Education, Universidad Catolica de la Santísima Concepcion, Concepción, Chile; ^4^ Division of Sport and Exercise Sciences, School of Applied Sciences, Abertay University, Dundee, United Kingdom; ^5^ Sport Performance Research Institute New Zealand (SPRINZ), Auckland University of Technology, Auckland, New Zealand; ^6^ Department of Physical Education and Sport, Faculty of Sport Sciences, University of Granada, Granada, Spain

**Keywords:** fatigue, marathon athletes, repeated sprint ability, CrossFit, blood flow restriction (BFR)

**Editorial on the Research Topic**
Training optimization through the recording of neuromuscular variables

This editorial presents a collection of articles focused on the effective use of physiological and mechanical variables to assess athletes’ neuromuscular state and optimize their training programs. The included studies span a range of sports disciplines, from traditional activities like marathon running and sprinting to newer practices such as CrossFit. Additionally, the editorial features a meta-analysis on the effects of blood flow restriction on muscle activation and post-activation performance enhancement during actions involving the upper-body muscles.

The study by Cosio et al. explores the challenges of recovery following repeated-sprint training, a common regimen in many high-intensity sports. The authors specifically focused on the hamstring muscles, which are highly susceptible to injury during eccentric contractions in the late swing phase. By examining inter-individual variability in recovery among male and female athletes, the authors offer valuable insights into the nuances of muscle recovery and the biomarkers associated with muscle damage.

Cosio et al. highlight the importance of individualized recovery protocols, revealing that some athletes experience significant and prolonged reductions in force-generating capacity and range of motion following a repeated spring training. These findings highlight the potential dangers of a one-size-fits-all approach to training recovery. The 90° hip: 20° knee test emerges as a cost-effective tool for monitoring hamstring recovery, where reductions in maximal voluntary isometric contraction torque persisted up to 72 h post-exercise. The study of Cosio et al. also identifies limitations in current assessment tools, particularly for female athletes. Specifically, the Jurdan test, commonly used to monitor hamstring recovery, was less effective in detecting post-exercise reductions in muscle function in women. This reduced effectiveness can be attributed to higher stretch tolerance, different joint geometry, and lower passive stiffness in female compared to male athletes.

Another study featured in this editorial, led by Nescolarde et al., also focused on the hamstring muscles, aiming to find a non-invasive, easily measurable parameter for predicting marathon race times. The study examines the relationship between serum biomarkers, such as C-reactive protein and creatine kinase, and localized bioimpedance parameters in recreational athletes. Their findings reveal that bioimpedance reactance and phase angle measurements may predict marathon performance, correlating with race time and muscle damage markers. This study introduces localized bioimpedance parameters as a useful tool for monitoring muscle condition and fatigue, providing athletes and coaches with actionable data to adjust training loads and recovery strategies.

High-intensity fitness programs like CrossFit have gained global popularity for enhancing performance and overall health. These programs combine various training modalities, including weightlifting, gymnastics, calisthenics, and resistance exercises, creating diverse and intense workouts. However, the acute physiological effects of these workouts are not well understood. In this collection of studies, Barba-Ruíz et al. examined the acute effects of various CrossFit modalities on heart rate, mean propulsive velocity (MPV), and intra- and inter-set MPV loss. Specifically, the three explored CrossFit modalities were (i) “As Many Rounds as Possible” (AMRAP), where participants performed 10 repetitions of each exercise within 12 min at their own pace, (ii) “Every Minute on the Minute” (EMOM), where participants completed 10 repetitions each minute, resting until the minute expires, and (iii) “Rounds for Time” where participants completed half the rounds achieved in AMRAP as quickly as possible. The findings showed that each modality induced different levels of fatigue. For instance, AMRAP resulted in lowest absolute MPV values due to its self-regulated pacing, “Rounds for Time”, which encourages athletes to complete the workout as quickly as possible, led to higher MPV losses and greater fatigue, while EMOM resulted in the lowest heart rate, making it suitable for sustained effort. These insights emphasize the need to choose training modalities that align with the athlete's specific goals—whether that is maximizing power output, sustaining effort, or enhancing technique maintenance under fatigue.

Finally, the meta-analysis by Liu et al. included in this collection offers a comprehensive evaluation of the impact of blood flow restriction training (BFRT) on muscle activation and post-activation potentiation in the upper limbs. BFRT has gained popularity for its ability to enhance muscle strength and hypertrophy at lower loads, making it particularly useful in rehabilitation and performance enhancement contexts. Liu et al. reviewed 31 randomized controlled trials and found strong evidence that BFRT significantly enhances muscle activation, as reflected by increased integrated electromyography values and improves “explosive” force in the upper limbs. The analysis suggests that BFRT is most effective when applied with exercise intensities ranging from 40% to 70% of one-repetition maximum and pressure intensities of 60% or more of arterial occlusion pressure. By elucidating the factors that influence BFRT's effectiveness, Liu et al. offer valuable insights that can assist athletes and coaches in tailoring their training strategies to maximize performance benefits.

The collection of articles in this editorial emphasizes the critical role of monitoring neuromuscular variables in optimizing training programs across various sports disciplines. The research highlights the importance of tailored, data-driven approaches to enhancing athletic performance and emphasizes the potential of innovative measurement techniques and training methods. Together, these studies provide valuable insights into the development of more effective and efficient training protocols, ultimately contributing to improved athlete outcomes and advancements in sports science.

